# Dietary intake of live microbes is inversely associated with fatigue and modified by serum folate among adults aged 40 years or more

**DOI:** 10.1016/j.maturitas.2026.108868

**Published:** 2026-02-03

**Authors:** Galya Bigman, Amber S. Kleckner, Yuanyuan Li, Elizabeth A. Dennis, Alice S. Ryan, John D. Sorkin

**Affiliations:** a Division of Gerontology, Department of Epidemiology and Public Health, University of Maryland School of Medicine, Baltimore, MD, 21201, USA; b Department of Pain and Translational Symptom Science, University of Maryland School of Nursing, Baltimore, MD, 21201, USA; c Department of Nutrition and Food Science, University of Maryland, College Park, MD, 20742, USA; d Department of Physical Therapy and Rehabilitation Science, University of Maryland School of Medicine, Baltimore, MD, 21201, USA; e Geriatric Research, Education, and Clinical Center (GRECC), Veterans Affairs Maryland Health Care System, Baltimore, MD, 21201, USA; f Division of Gerontology, Geriatrics, and Palliative Medicine, Department of Medicine, University of Maryland School of Medicine, Baltimore, MD, 21201, USA

**Keywords:** Fatigue, Live microorganisms, Folate, Diet quality, Aging, NHANES

## Abstract

**Objectives:**

To investigate the independent associations between dietary live-microbe intake, as well as circulating levels of folate metabolites, and fatigue, and to examine their interaction as a potential biological pathway underlying fatigue in aging adults.

**Study design:**

Cross-sectional analysis of adults aged ≥40 years participating in the National Health and Nutrition Examination Survey 2011–2023.

**Main outcome measures:**

Fatigue was assessed using the Patient Health Questionnaire fatigue item and categorized as none/low versus moderate/severe. Dietary intake of live-microbe foods was derived from two 24-h recalls and classified as low, medium, or high. Total serum concentrations folate and its metabolites, including 5-methyltetrahydrofolate, were quantified. Survey-weighted logistic regression, adjusted for sociodemographic, dietary, and clinical covariates, estimated main effects and interactions between the effects of 5-methyltetrahydrofolate and of live-microbe intake; sensitivity analyses additionally adjusted for depressive symptoms and sleep disturbance.

**Results:**

Moderate/severe fatigue was reported by 16.3% of 14,376 participants and 15.0% reported no intake of live-microbe foods. High versus low live-microbe intake was associated with lower odds of moderate/severe fatigue (odds ratio [OR] 0.60; 95% confidence interval [CI] 0.46–0.79). Higher serum 5-methyltetrahydrofolate levels were also associated with lower odds of moderate/severe fatigue (OR 0.85; 95% CI 0.73–0.98). In stratified analyses, high live-microbe intake corresponded to 38–65% lower odds of moderate/severe fatigue only among adults with higher levels of 5-methyltetrahydrofolate, with no significant associations among those with lower levels.

**Conclusions:**

Intake of microbe-rich foods and higher levels of circulating 5-methyltetrahydrofolate are both associated with lower levels of fatigue in midlife and older adults, and folate sufficiency appears to potentiate the fatigue-reducing benefits of live-microbe foods, supporting a nutrient–microbe pathway relevant to healthy aging.

## 1. Introduction

Fatigue represents a prevalent and inherently multidimensional symptom, marked by persistent exhaustion, diminished energy reserves, and compromised physical or cognitive performance that is not adequately alleviated through rest [1,2]. Epidemiological estimates suggest that roughly one in five adults experience some level of fatigue, a condition that can erode functional capacity, undermine quality of life, and generate significant societal costs, exceeding $130 billion annually in lost productivity in the United States alone [3]. The burden of fatigue is disproportionately high among individuals with chronic illnesses such as cancer and cardiovascular disease and has been further amplified by the emergence of long COVID associated sequelae [4,5]. Notably, fatigue is also reported in ostensibly healthy adults, where it may serve as an early marker of physiological vulnerability [6]. Fatigue acquires greater clinical and public health relevance in midlife and later adulthood, when age related reductions in metabolic efficiency and nutrient absorption, combined with diminished physiological resilience and higher rates of chronic disease, contribute to sustained fatigue [6].

Diet is increasingly recognized as a potential contributor to fatigue, although the biological pathways linking dietary exposures to energy regulation remain incompletely understood [7,8]. Insufficient intake of key micronutrients, including B vitamins, vitamin C, iron, magnesium, and zinc, may compromise mitochondrial function and cellular energy production, whereas frequent consumption of ultra-processed foods has been associated with systemic inflammation, metabolic dysregulation, and unfavorable changes in body composition [7,8]. Such dietary patterns also shape the gut microbiome, contributing to reductions in beneficial microbial taxa, greater intestinal permeability, impaired nutrient absorption, and altered immune responses [9–11]. Conversely, diets that foster microbial diversity may support more stable metabolic homeostasis, yet their specific relevance to the experience of fatigue has not been clearly established [12,13].

Folate (vitamin B9) plays a key role in one-carbon metabolism, methylation, nucleotide synthesis, and mitochondrial energy production, and interacts closely with microbial pathways involved in folate synthesis and utilization [14,15]. Both dietary and microbially derived folate contribute to circulating folate pools, particularly tetrahydrofolate (THF) and 5-methyltetrahydrofolate (5-MTHF), which are essential for cellular and neural energy regulation [14,15]. Folate metabolism supports DNA synthesis, repair, and methylation through the folate cycle and closely interacts with vitamin B6 and B12 to support red erythropoiesis and cellular energy processes [17]. Disruptions in folate-dependent pathways have been linked to impaired mitochondrial function, neurobehavioral symptoms, and fatigue [7,16,17]. In older adults, age-related changes in dietary intake and gastrointestinal function may further impair folate absorption, leading to anemia, reduced oxygen delivery and cellular energy efficiency, which can manifest as chronic fatigue in this population [6,16,17].

Foods containing live microorganisms, such as yogurt, kefir, kimchi, fermented vegetables, and raw fruits and vegetables, introduce beneficial microbes that enhance microbial diversity and support key metabolic processes, including short-chain fatty acid and B-vitamin production [18]. Higher intake of these foods has been linked to improved age-related health outcomes [19,20]; however, whether these dietary patterns relate specifically to fatigue, and whether any such associations are influenced by metabolic factors such as folate status, remains unknown.

We evaluated the prevalence of fatigue among U.S. adults aged ≥40 years and investigated whether habitual intake of live-microbe foods and circulating folate metabolites were associated with fatigue. We further examined folate status as a potential modifier of these associations, anticipating that greater microbial and folate exposures would each be inversely associated with fatigue and that folate sufficiency would enhance the live-microbe–fatigue relationship.

## 2. Methods

### 2.1. Study population

The National Health and Nutrition Examination Survey (NHANES) is a continuous, cross-sectional program designed to assess the health and nutritional status of the U.S. civilian, non-institutionalized population [21]. Data are collected through household interviews, standardized physical examinations, and laboratory assessments conducted in mobile examination centers. Because of the cross-sectional design, analyses are intended to evaluate associations and do not permit causal inference. All protocols were approved by the National Center for Health Statistics Ethics Review Board, and written informed consent was obtained from all participants [22].

NHANES cycles from 2011 to 2016, 2017–March 2020, and 2021–2023 were pooled following National Center for Health Statistics (NCHS) guidance [23]. Because dietary intake of live microbes was the primary exposure of interest, primary analyses used the two-day dietary recall examination weights (WTDR), rescaled to account for unequal cycle lengths by the proportion of survey years contributed by each period (2011–2016: 1/6; 2017–March 2020: 2/6; 2021–2023: 1/6). The combined survey design incorporated the rescaled weights, masked variance strata, and primary sampling units using Taylor series linearization. Handling of the partial 2017–March 2020 cycle and the 2021–2023 pandemic and post-pandemic cycles followed NCHS recommendations for combining pre- and post-pandemic NHANES data. Associations involving serum folate biomarkers were additionally examined under alternative weighting schemes for applicable cycles to confirm robustness of effect estimates [23]. Participants aged 40 years and older were eligible for inclusion. Individuals were excluded if they were missing dietary recall data, serum folate metabolite measurements, fatigue responses, or required covariates, or if they reported energy intake below 500 or above 5000 kcal/day.

### 2.2. Fatigue assessment

Fatigue was measured using the Patient Health Questionnaire (PHQ) item, *“Over the last two weeks, how often have you been bothered by feeling tired or having little energy?”* Response options included 0 = not at all, 1 = several days, 2 = more than half the days, and 3 = nearly every day. Consistent with prior epidemiologic studies [24], responses were dichotomized as none/low (0–1) versus moderate–severe (2–3).

### 2.3. Dietary intake of live microbes

Dietary data were obtained from up to two interviewer-administered 24-h dietary recalls, with intake reported by participants using the Automated Multiple-Pass Method. Day 1 recalls were conducted in person and Day 2 by telephone 3–10 days later. All foods and beverages were coded with USDA Food and Nutrient Database for Dietary Studies food codes [25]. Each item was classified using the Classification System for Defining and Estimating Dietary Intake of Live Microbes [26,27] as “low” (<10^4^ CFU/g), “medium” (10^4^–10^7^ CFU/g), or “high” (>10^7^ CFU/g) in viable microbial content. In general, foods subjected to pasteurization or high-temperature processing (e.g., most cooked foods, refined grains, and shelf-stable products) were classified as “low” in live microbes. “Medium” live-microbe foods primarily included unpeeled fresh fruits and vegetables and minimally processed plant foods. High live-microbe foods consisted mainly of unpasteurized or fermented products, such as yogurt, fermented dairy products, and other foods produced through microbial fermentation.

Referring to prior literature, for each 24-h dietary recall, intake of live-microbe foods was categorized hierarchically as high (any high-microbial food), medium (≥1 medium-microbial food and no high-microbial foods), or low [26–28]. This hierarchical classification prioritizes the presence of high-microbial foods, reflecting their disproportionate contribution to total microbial exposure. For participants with two dietary recalls, six intake patterns were derived (L–L, L–M, L–H, M–M, M–H, H—H). In parallel, a semi-quantitative MedHi Index was constructed based on total gram intake of medium- and high-microbial foods, following Gille et al. [27], categorizing individuals as G1 (none), G2 (>0 to <median), or G3 (≥median).

### 2.4. Serum folate metabolites

Beginning in 2011–2012, NHANES quantified six serum folate metabolites using isotope-dilution liquid chromatography–tandem mass spectrometry at the CDC Nutritional Biomarkers Laboratory: 5-methyltetrahydrofolate (5-MTHF), unmetabolized folic acid, tetrahydrofolate, 5-formyltetrahydrofolate, 5,10-methenyl-tetrahydrofolate, and MeFox (oxidized 5-MTHF). Total serum folate was calculated as the sum of all metabolites. Values below the lower limit of detection were imputed as LOD/√2 per CDC protocols [29]. All folate variables were natural-log–transformed prior to analysis.

### 2.5. Covariates

According to prior NHANES literature, covariates included age, sex, race/ethnicity, education, marital status, BMI category, chronic disease status (CVD, COPD, cancer, diabetes), alcohol intake, diet quality (NRF9.3) [31], and total energy intake. These variables were pre-specified based on prior literature and biological plausibility, reflecting established associations with diet, folate metabolism, and fatigue [26–34]. Depressive symptoms and sleep disturbance were derived from PHQ-9 items, with core depressive symptoms defined by affective and cognitive items (items 1, 2, and 6–9) and sleep disturbance defined by item 3 (trouble sleeping or sleeping too much); both were dichotomized as scores of 0–1 versus ≥2. Definitions, NHANES variables, and coding for all covariates are provided in Supplementary Table 1.

### 2.6. Statistical analysis

All analyses incorporated NHANES examination weights, strata, and primary sampling units to account for the complex survey design. Weighted means, proportions, and percentile estimates were used to describe sample characteristics, with group differences assessed using survey-weighted Wald F tests and χ^2^ tests. A core set of covariates was pre-specified a priori [26–35]. Statistical screening using a liberal threshold (*p* < 0.20) in bivariate analyses was used only as a supplementary diagnostic to evaluate potential residual confounding and did not determine inclusion in the primary multivariable models. Survey-weighted logistic regression was used to examine associations of (1) dietary intake of live microbes and (2) serum folate metabolites (log-transformed) with fatigue. Each exposure was evaluated in crude model (unadjusted), sociodemographic-adjusted, and fully adjusted models (including BMI, chronic disease status, alcohol intake, total energy intake, and NRF9.3 diet quality). Effect modification by serum folate was assessed using multiplicative interaction terms (dietary intake of live microbes × serum folate), with serum folate categorized by median split. Significant interactions (*p* < 0.05) were probed using stratified analyses.

Sensitivity analyses separately accounted for depressive symptoms and sleep disturbance to reduce potential structural confounding arising from overlap between affective symptom measurement and the fatigue outcome. An additional sensitivity analysis incorporated total dietary folate intake into the fully adjusted interaction models used in the primary analysis, to evaluate potential confounding by folate consumption. All statistical analyses were conducted using Stata 19.5 with two-sided *p* < 0.05 considered statistically significant. Fig. 3 was generated in RStudio using base R plotting functions.

## 3. Results

### 3.1. Participant selection and analytic sample

**Fig. 1.**
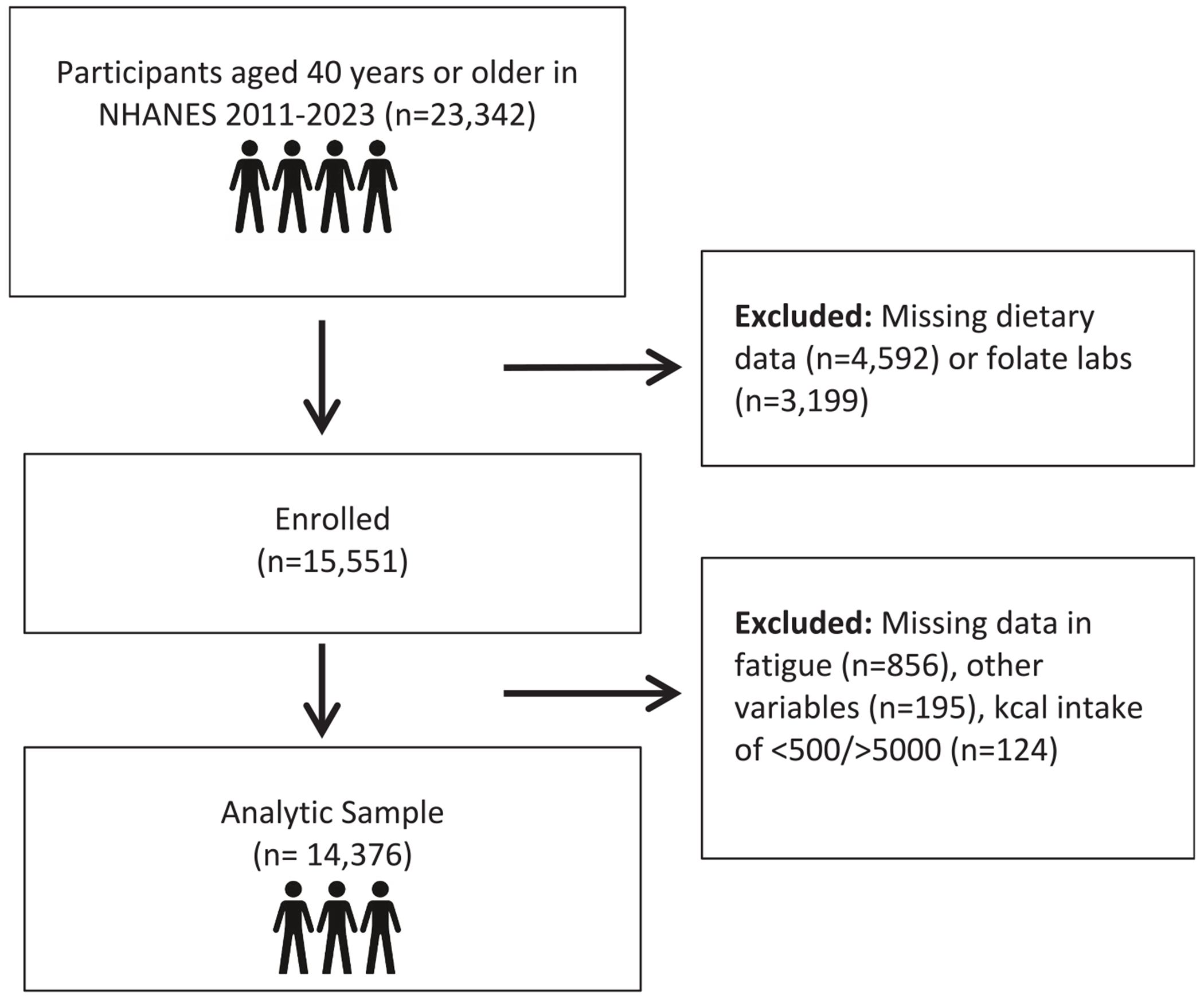
Flowchart of participant inclusion and exclusion for the analytic sample, NHANES 2011–2023.

A total of 23,342 adults aged 40 years and older were identified across NHANES 2011–2023. After excluding participants missing dietary data (*n* = 4592), serum folate metabolite measurements (*n* = 3199), fatigue information (*n* = 856), or required covariates (*n* = 195), 14,376 individuals remained for the analytic sample (Fig. 1).

Participants in the analytic sample were slightly younger and healthier, but distributions of fatigue, dietary intake of live microbes, and other covariates were similar to excluded cases, indicating no major systematic differences by missingness (Supplementary Table 2).

### 3.2. Participant characteristics by fatigue

Among 14,376 adults aged 40 years and older, 2500 (16.3%) reported fatigue. Compared with those reporting none or low fatigue, adults with fatigue were more often female and had lower educational attainment (*p* < 0.001). They also had higher rates of obesity and chronic conditions such as cardiovascular disease, COPD, and diabetes (p < 0.001). Diet quality, total energy intake, and total folate intake were lower in the fatigue group, and nondrinking was more common (p < 0.001). Mean age and racial and ethnic distribution did not differ significantly between groups (*p* > 0.05) (Table 1).

### 3.3. Distributions of dietary intake of live microbes and folate metabolites by fatigue

Overall, 15.0% reported no intake of live-microbe foods. Among high live-microbe foods, intake was dominated by fermented dairy products, with cheese accounting for approximately 56% and yogurt for approximately 33% of intake events. Medium live-microbe foods were primarily plant-based, consisting mainly of vegetables (approximately 53%) and fruits (approximately 28%), with additional contributions from condiments and sauces (approximately 10%). Low live-microbe foods were broadly distributed across pasteurized and processed food groups.

**Fig. 2.**
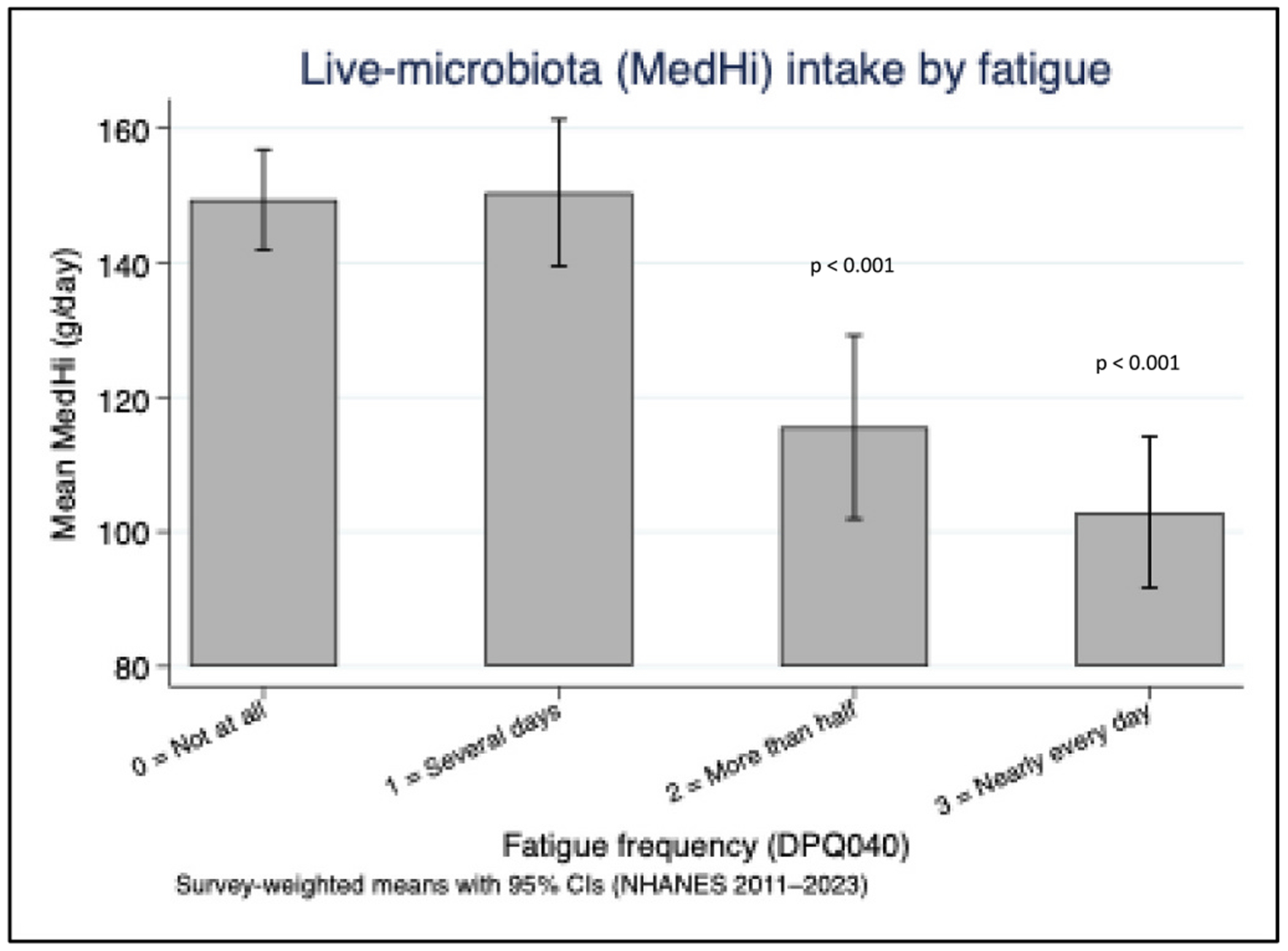
Survey-weighted mean MedHi intake (g/day) by fatigue status among U.S. adults aged ≥40 years, NHANES 2011–2023. Mean intake of medium- and high-microbial foods (MedHi Index) is displayed by fatigue category (none/low vs. moderate–severe). Error bars show 95% confidence intervals based on survey-weighted estimates.

Adults with fatigue were more likely to fall into lower intake categories, particularly L–L and L–M, compared with those reporting none or low fatigue (*p* < 0.001). Higher intake categories (H—H and M–H) were less common among participants with fatigue. Similar patterns were seen with the MedHi Index: individuals with fatigue more frequently fell into groups G1 and G2 compared with those without fatigue (*p* < 0.001) (Supplementary Table 3). As shown in Fig. 2, mean MedHi intake declined steadily as fatigue frequency increased (p < 0.001).

5-MTHF differed significantly by fatigue status (Supplementary Table 4). Adults with fatigue had lower 5-MTHF (median 34.2 [21.8–52.8] nmol/L) compared with those with none or low fatigue (37.8 [24.5–56.3] nmol/L; *p* = 0.008). Total serum folate showed a similar but nonsignificant trend (*p* = 0.060). Other metabolites, including folic acid, THF, 5-formyl-THF, 5,10-methenyl-THF, and RBC folate, did not differ by fatigue status (all *p* > 0.05). MeFox levels were significantly higher among adults with fatigue (*p* < 0.001).

### 3.4. Association between dietary intake of live microbes and fatigue

Fatigue prevalence in the study population was 16.3%, therefore, odds ratios should be interpreted as measures of relative odds rather than risk; accordingly, the reported effect sizes may modestly overestimate the corresponding risk ratios. Higher dietary intake of live microbes was associated with lower odds of fatigue in a dose–response pattern after full adjustment (Table 2). Compared with L–L, intermediate intake patterns showed 20–40% lower odds of fatigue (OR range: 0.66–0.80), and consistently high intake (H—H) was associated with a 40% reduction (OR = 0.60, 95% CI: 0.46–0.79; p-trend <0.001). These findings indicate a graded inverse association between habitual intake of live-microbe foods and fatigue. The MedHi Index produced comparable findings: fatigue prevalence decreased progressively from G1 to G3 (G3 OR = 0.67, 95% CI: 0.53–0.84; p-trend = 0.002). In practical terms, the observed odds ratios correspond to approximately 20–40% lower odds of having fatigue among adults reporting regular consumption of foods containing live microorganisms, and approximately 40% lower odds among those with consistent intake of high–microbial foods such as fermented dairy products (e.g., yogurt, kefir), fermented vegetables (e.g., kimchi, sauerkraut), and other traditionally fermented foods.

### 3.5. Associations between serum folate metabolites and fatigue

Among folate metabolites, serum 5-MTHF remained inversely associated with fatigue after full adjustment (OR = 0.85, 95% CI: 0.73–0.98; *p* = 0.024). Total serum folate showed borderline significance (*p* = 0.061). No significant associations were observed for folic acid or MeFox after adjustment (Table 3). Given its significance, only 5-MTHF was included in effect-modification analyses.

### 3.6. Serum 5-MTHF modifies the association between dietary intake of live microbes and fatigue

**Fig. 3.**
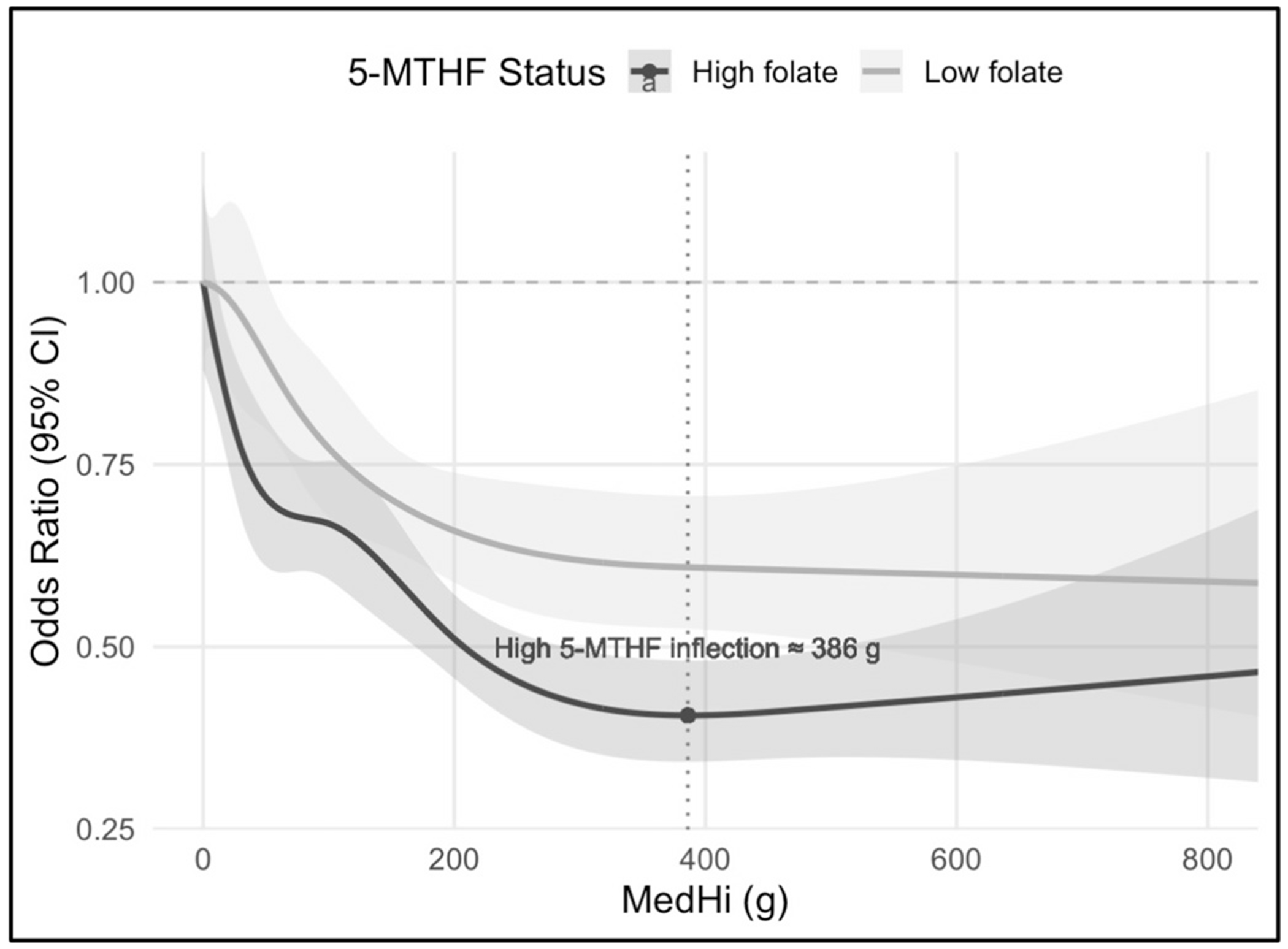
Dose–response association between MedHi intake and fatigue stratified by 5-methyltetrahydrofolate (5-MTHF). Survey-weighted logistic regression curves showing the adjusted odds of moderate–severe fatigue across increasing MedHi intake (g/day), stratified by low versus high serum 5-MTHF. Shaded areas represent 95% confidence intervals.

Serum 5-MTHF, categorized by a median split, significantly modified the association between dietary intake of live microbes and fatigue (interaction *p* = 0.027 for the six-level measure and *p* = 0.012 for MedHi; Table 4). Among adults with higher 5-MTHF, dietary intake of live microbes showed strong, graded inverse associations with fatigue (H—H vs. L–L: OR = 0.35, 95% CI: 0.23–0.54). MedHi results were similar. No significant associations were observed among adults with 5-MTHF below the median levels. Among adults with high 5-MTHF, above the median, increasing MedHi intake produced a decline in fatigue risk, reaching OR ≈ 0.4 around 386 g/day of foods containing live microorganisms. In the low-5-MTHF group, inverse associations persisted but were weaker, with ORs ≈ 0.6–0.7 across most of the intake range (Fig. 3).

As a robustness check, effect modification was additionally modeled using a continuous interaction between log-transformed 5-MTHF and live microbiota intake, yielding results consistent with the primary categorical interaction (overall interaction *p* = 0.006).

### 3.6.1. Sensitivity analyses adjusting for depressive symptoms and sleep disturbance

Elevated sleep disturbance and elevated core depressive symptoms were present in 15.8% and 27.0% of the population, respectively. Adjustment for core depressive symptoms modestly attenuated the associations between live microbiota intake and fatigue; however, overall associations remained statistically significant when modeled using six intake categories and the MedHi Index (overall *p* = 0.008 and *p* = 0.007, respectively; Supplementary Table 5). In addition, adjustment for sleep disturbance attenuated the associations toward the null when modeled using six intake categories (overall *p* = 0.054), while associations remained statistically significant when intake was modeled using the MedHi Index (overall *p* = 0.048; Supplementary Table 6).

### 3.6.2. Sensitivity analysis adjusting for dietary folate

High dietary intake of live microbes (H—H) had greater dietary folate intake than low dietary intake of live microbes (L–L) (516 ± 252 μg vs. 435 ± 260 μg; *p* < 0.001); data not shown. Therefore, because foods containing live microorganisms can also contribute dietary folate, total folate intake was added to the fully adjusted interaction models After adjustment, the interaction remained significant for both dietary intake of live microbes (*p* = 0.033) and the MedHi Index (*p* = 0.032), indicating that dietary folate did not account for the observed effect modification.

## 4. Discussion

In this nationally representative sample of U.S. adults aged 40 years and older, higher dietary intake of live microbes and higher circulating folate metabolites, particularly 5-MTHF, were each inversely associated with fatigue. To our knowledge, this is the first population-based study to evaluate whether dietary intake of live microbes and circulating folate metabolites jointly relate to fatigue, providing new evidence for a potential nutrient–microbe interaction in energy regulation. Notably, the association between dietary live-microbe intake and fatigue was present primarily among adults with higher serum 5-MTHF, suggesting a nutrient–microbe interaction relevant to energy regulation [16,17]. Because 5-MTHF is the predominant bioactive folate form and a central participant in one-carbon metabolism, methylation, and mitochondrial energy processes, individuals with greater functional folate availability may be more responsive to microbial exposures that influence metabolic and immune pathways [14,20]. The absence of association among those with low serum folate levels further suggests that microbial intake alone may be insufficient when folate-dependent pathways are constrained.

While total serum folate showed an inverse crude association with fatigue, this relationship attenuated after adjustment, indicating that the observed effect is more strongly linked to biologically active 5-MTHF than to the overall folate pool or its oxidative metabolites. The lack of association for other folate metabolites and RBC folate aligns with the view that recent, functional folate availability, rather than long-term folate stores, is more relevant to fatigue perception [32]. Serum 5-MTHF reflects short-term folate intake and microbial folate contributions, whereas RBC folate reflects cumulative folate exposure over the erythrocyte lifespan and may be less sensitive to microbially derived folate synthesized in the gut [20,32].

Mechanistically, 5-MTHF is the primary circulating methyl donor supporting methionine and S-adenosylmethionine (SAM) synthesis [16,17,29], implicating methylation-related processes in pathways regulating fatigue [14,32,33]. Variability in 5-MTHF within normal physiological ranges may influence mitochondrial function, neurotransmitter metabolism, inflammatory signaling, and epigenetic regulation through SAM-dependent one-carbon pathways [16,17,32], mechanisms that have been linked to fatigue across clinical and nonclinical populations [7,8,33]. The finding that 5-MTHF, but not 5′,10′-methylene THF, was associated with fatigue supports the hypothesis that methylation-related pathways, rather than nucleotide synthesis, are more relevant to energy-related symptoms.

Alterations in the gut microbiome have been linked to fatigue-related conditions, including chronic fatigue syndrome [9], long-COVID [4,5], and cancer-related fatigue through nutrition-metabolic pathways [24]. Mechanistic studies indicate that a balanced, SCFA-producing microbiome may influence host physiology through SCFA generation, intestinal barrier integrity, and inflammatory regulation [10–13,35], while dietary live microbes can modulate gut microbial activity [18,26,27] and contribute to microbial synthesis of B vitamins, including folate [14,15]. Our findings extend this literature by identifying folate status as a potential modifier of the association between dietary microbial exposure and fatigue, consistent with evidence that host folate pathways and one-carbon metabolism are implicated in fatigue biology [16,17,29,33]. These results suggest a synergistic nutrient–microbe pathway that has not previously been demonstrated in population-based research.

From a clinical and public health perspective, these findings suggest that dietary strategies aimed at maintaining folate sufficiency while promoting gut microbial diversity, such as increased intake of folate-rich foods and foods containing live microorganisms, may represent feasible lifestyle approaches to mitigate fatigue. The observed nutrient–microbe interaction highlights a biologically plausible target for future dietary and lifestyle interventions, including personalized nutrition approaches based on folate status and microbial profile.

This could be explored in future studies using controlled feeding trials to determine the optimal dosage and source of live-microbe foods required to achieve clinically meaningful improvements in patient-reported fatigue [18,26,27], while also controlling for intake of other SCFA-promoting foods that may influence microbiome-derived metabolites [12,13]. Future research should assess broader symptom profiles, health-related quality of life, and physical function [1,6], and further examine the interaction between live-microbe intake and folate status [14–17,29,33]. Such studies would benefit from more comprehensive assessments of fatigue, dietary intake, and folate biomarkers in clinical settings [1,25,29].

This study has several strengths. The large, nationally representative sample enhances generalizability. Comprehensive profiling of serum folate metabolites, including six individual forms, provided a nuanced view of one-carbon metabolism relevant to fatigue. The dual-method approach to estimating dietary live-microbe intake captured both categorical exposure patterns and dose–response gradients. Effectmodification analyses provided mechanistic insight, linking dietary microbial intake and fatigue within the context of folate availability.

Several limitations should be considered. Fatigue was assessed using a single PHQ item, which may introduce nondifferential misclassification; however, single-item fatigue measures are commonly used and validated in epidemiologic research [25]. Fatigue is multidimensional, influenced by physical, psychological, and social factors, and our measure captures perceived energy rather than specific etiologies. Dietary microbial intake was estimated from food recalls using expertbased classifications rather than direct microbial quantification, which may introduce measurement error. Because the study design is crosssectional, causality cannot be inferred; reverse causation is possible if fatigued individuals reduce consumption of microbe-rich foods. Finally, despite adjustment for multiple confounders, including sensitivity analyses for sleep and depression, residual confounding and unmeasured factors cannot be excluded, such as overall dietary quality based on food intake, anemia, sleep patterns, and psychosocial stress [1,6,24,31].

## 5. Conclusion

This study provides the first population-based evidence that the relationship between dietary intake of live microbes and fatigue differs by serum folate status in midlife and older adults. Individuals with higher functional folate availability, particularly circulating 5-MTHF, appeared more responsive to the potential benefits of microbe-rich foods, suggesting a nutrient–microbe interaction relevant to energy regulation. These findings highlight a previously unrecognized link between folate-dependent metabolic processes and habitual microbial exposure. Future mechanistic and intervention studies are needed to determine whether improving dietary live-microbe intake, enhancing folate status, or targeting both pathways can support energy balance and reduce fatigue, particularly in populations vulnerable to post-viral or age-related fatigue. These findings support probiotic intervention trials that evaluate changes in circulating 5-methyltetrahydrofolate as a key mechanistic mediator to optimize efficacy and identify populations most likely to benefit, including larger and more diverse aging cohorts.

## Supplementary Material

Supplementary

## Figures and Tables

**Table 1:** Weighted sociodemographic, lifestyle, and health characteristics of U.S. adults aged ≥40 years, by fatigue status.

		Fatigue		
Characteristics	Total N (%) 14,376 (100.0)	None/low N (%) 11,876 (83.7)	Moderate/Severe N (%) 2500 (16.3)	p-value
Age, years, mean ± SD	58.3 ± 11.5	58.2 ± 11.4	58.7 ± 11.7	0.145
Sex				< 0.0001
Male	6770 (46.8)	5831 (48.8)	939 (36.4)	
Female	7606 (53.2)	6045 (51.2)	1561 (63.6)	
Race/Ethnicity				0.046
NH White	6698 (70.0)	5479 (70.4)	1219 (68.0)	
NH Black	2917 (10.0)	2370 (9.6)	547 (12.0)	
Others	4761 (20.0)	4027 (20.0)	734 (20.0)	
Education				< 0.001
Less than HS	6025 (35.6)	4790 (33.7)	1235 (45.1)	
Education (HS Grad)	4283 (30.7)	3480 (30.1)	803 (33.6)	
More than HS	4068 (33.8)	3606 (36.2)	462 (21.3)	
Marital Status				< 0.001
Married/living with partner	8753 (67.2)	7497 (69.2)	1256 (57.1)	
Formerly	4842 (28.6)	3762 (26.7)	1080 (38.2)	
Never	781 (4.2)	617 (4.1)	164 (4.7)	
BMI (kg/m^2^)†				< 0.001
Normal	3380 (23.3)	2902 (24.0)	478 (19.6)	
Overweight	4884 (34.5)	4197 (36.1)	687 (26.5)	
Obese	6112 (42.7)	4777 (39.9)	1335 (53.9)	
Chronic Disease				
CVD	2164 (12.7)	1556 (10.9)	608 (21.9)	< 0.001
COPD	861 (6.0)	570 (4.8)	291 (12.1)	< 0.001
Cancer	2140 (16.1)	1709 (15.7)	431 (18.1)	0.078
Diabetes	2660 (15.0)	2037 (13.9)	623 (20.3)	< 0.001
≥1 of the above (Yes)	5740 (36.6)	4421 (34.4)	1319 (49.3)	< 0.001
Diet Quality (NRF9.3)				< 0.001
T1-Low	4305 (29.5)	3391 (27.8)	914 (37.9)	
T2-Medium	4882 (33.8)	4044 (33.9)	838 (33.7)	
T3-High	5189 (36.7)	4441 (38.3)	748 (28.4)	
Energy Intake (Kcal)	2016 ± 729	2030 ± 720	1947 ± 772	0.001
Total Folate Intake (μg/day)	479 ± 263	486 ± 265	440 ± 247.	< 0.001
Alcohol Intake				< 0.001
Non-drinker (never/past)	5475 (30.7)	4377 (29.4)	1098 (37.1)	
Moderate (<2 drinks day)	3488 (27.6)	2953 (28.3)	535 (23.9)	
High (≥2 drinks day)	5413 (41.7)	4546 (41.3)	867 (39.0)	

Note. Values are survey-weighted and expressed as percentages for categorical variables or means ± SD for continuous variables. *p*-values are derived from the survey-weighted Wald F test (continuous) or Rao–Scott χ^2^ test (categorical). Abbreviations: NH, non-Hispanic; HS, high school; BMI, body mass index; NRF9.3, Nutrient Rich Food Index 9.3; Kcal, kilocalories; CVD, cardiovascular disease (including self-reported congestive heart failure, coronary heart disease, angina, heart attack, or stroke); COPD, chronic obstructive pulmonary disease (self-reported emphysema); “≥1 condition” indicates participants reporting one or more of these chronic diseases.

† Normal weight was defined as BMI 18.5–24.9 kg/m^2^; participants with BMI <18.5 kg/m^2^ (<5% of the sample) were included in this category. T, Tertile.

**Table 2:** Weighted logistic regression of dietary intake of live microbes and its association with fatigue among U.S. adults aged ≥40 years.

	Crude Model 1 OR (95% CI)	p-value	Adjusted Model 2 OR (95% CI)	p-value	Adjusted Model 3 OR (95% CI)	p-value
Dietary intake of live microbes †						
L-L	1.00		1.00		1.00	
L-M	0.73 (0.58–0.92)	0.007	0.77 (0.61–0.96)	0.023	0.80 (0.65–1.02)	0.078
L-H	0.58 (0.47–0.71)	<0.001	0.64 (0.51–0.79)	<0.001	0.71 (0.56–0.90)	0.005
M-M	0.60 (0.47–0.76)	<0.001	0.63 (0.49–0.81)	0.001	0.66 (0.51–0.85)	0.002
H-M	0.52 (0.41–0.65)	<0.001	0.61 (0.48–0.77)	<0.001	0.68 (0.54–0.88)	0.003
H-H	0.44 (0.34–0.57)	<0.001	0.52 (0.40–0.68)	<0.001	0.60 (0.46–0.79)	<0.001
P-trend		<0.001		<0.001		<0.001
Overall		<0.001		<0.001		0.002
P-value						
MedHi Index ††						
G1	1.00		1.00		1.00	
G2 (>0 & < median)	0.69 (0.57–0.82)	<0.001	0.73 (0.62–0.88)	0.001	0.76 (0.62–0.92)	0.005
G3 (≥ median)	0.51 (0.41–0.62)	<0.001	0.58 (0.47–0.72)	<0.001	0.67 (0.53–0.84)	0.001
P-trend		<0.001		<0.001		0.002
Overall		<0.001		<0.001		0.003
P-value						

Note. Values are survey-weighted odds ratios (ORs) and 95% confidence intervals (CIs) from logistic regression models. Given that fatigue prevalence was approximately 16.3%, ORs should be interpreted as measures of relative odds and may modestly overestimate the corresponding risk ratios; they should not be interpreted as risk ratios or absolute measures of effect. Model 2 adjusted for age, sex, race/ethnicity, education, and marital status. Model 3 further adjusted for BMI category, chronic disease status, NRF9.3 diet quality, alcohol intake, and total energy intake. p-values correspond to survey-adjusted Wald tests for linear trend or overall group differences.

† Categories (L–L, L–M, M–M, H–L, H–M, H–H) represent all combinations of low (L), medium (M), and high (H) microbial-rich food intake across two 24-h dietary recalls.

†† The MedHi Index provides an alternative semi-quantitative measure based on total gram intake of medium- and high-microbial foods (G1 = none, G2 = below median, G3 = above median among consumers).

**Table 3:** Weighted logistic regression of serum folate metabolites and their association with fatigue among U.S. adults aged ≥40 years.

	Crude Model 1 OR (95% CI)	p-value	Adjusted Model 2 OR (95% CI)	p-value	Adjusted Model 3 OR (95% CI)	p-value
Serum Folate Metabolites(nmol/L)						
Total folate	0.80 (0.71–0.92)	0.001	0.81 (0.70–0.93)	0.002	0.87 (0.76–1.00)	0.061
5-methyl-THF	0.77 (0.68–0.88)	<0.001	0.78 (0.68–0.90)	0.001	0.85 (0.73–0.98)	0.024
Folic acid	1.08 (1.00–1.17)	0.048	1.06 (0.97–1.15)	0.208	1.06 (0.98–1.16)	0.165
MeFox (oxidized)	1.15 (1.01–1.26)	0.017	1.13 (0.98–1.25)	0.047	1.05 (0.93–1.18)	0.428

Note. Values are survey-weighted odds ratios (ORs) and 95% confidence intervals (CIs) from logistic regression models. Given that fatigue prevalence was approximately 16.3%, ORs should be interpreted as measures of relative odds and may modestly overestimate corresponding risk ratios; they should not be interpreted as risk ratios or absolute measures of effect. Model 2 adjusted for age, sex, race/ethnicity, education, and marital status. Model 3 further adjusted for BMI category, chronic disease status, NRF9.3 diet quality, alcohol intake, and total energy intake. p-values correspond to survey-adjusted Wald tests. Folate metabolites were naturallog–transformed. Serum total folate represents the sum of all measured metabolites (5-methyl-THF, folic acid, and MeFox). Abbreviations: THF, tetrahydrofolate; MeFox, oxidized 5-methyl-THF.

**Table 4:** Weighted logistic regression of dietary intake of live microbes and fatigue, stratified by serum 5-methyl-THF level, among U.S. adults aged ≥40 years.

	5-methyl-THF			
	High (≥37.2)	*p*-value	Low (<37.2)	p-value
5-methyl-THF (nmol/L) Median [IQR]	55.70 [45.1–72.2]		24.00 [18.2–30.4]	<0.001
Dietary intake of live	OR (95% CI)		OR (95% CI)	
microbes †				
L-L	1.00	–	1.00	–
L-M	0.62 (0.42–0.91)	0.015	0.96 (0.769–1.31)	0.787
L-H	0.50 (0.35–0.71)	<0.001	0.95 (0.66–1.35)	0.776
M-M	0.51 (0.34–0.76)	0.001	0.78 (0.56–1.09)	0.156
H-M	0.52 (0.35–0.76)	0.001	0.82 (0.60–1.11)	0.196
H-H	0.35 (0.23–0.54)	<0.001	0.93 (0.67–1.30)	0.686
MedHi Index ††				
G1	1.00	–	1.00	–
G2 (>0 & < median)	0.60 (0.44–0.80)	0.001	0.88 (0.67–1.15)	0.335
G3 (≥ median)	0.45 (0.31–0.64)	<0.001	0.93 (0.69–1.25)	0.617

Note. Odds ratios (ORs) and 95% confidence intervals (CIs) were estimated from survey-weighted logistic regression models adjusted for age, sex, race/ethnicity, education, marital status, BMI category, chronic disease status, diet quality, alcohol intake, and total energy intake. Given that fatigue prevalence was approximately 16.3%, ORs should be interpreted as measures of relative odds and may modestly overestimate corresponding risk ratios; they should not be interpreted as risk ratios or absolute measures of effect. Serum folate metabolites were examined based on prior literature and biological plausibility. Reference categories were L–L for live-microbiota intake and G1 for the MedHi group. Serum folate status was categorized using a median split (low vs high). Interaction terms for live_microbiota6 × serum 5-methyl-THF and MedHi Index × serum 5-methyl-THF were statistically significant (p = 0.027 and p = 0.012, respectively).

Abbreviations: OR, odds ratio; CI, confidence interval; MedHi, medium- and high-microbial food index; 5-methyl-THF, 5-methyltetrahydrofolate.

† Categories (L–L, L–M, M–M, H–L, H–M, H–H) represent all combinations of low (L), medium (M), and high (H) microbial-rich food intake across two 24-h dietary recalls.

†† The MedHi Index provides a semi-quantitative measure based on total gram intake of medium- and high-microbial foods (G1 = none, G2 = below median, G3 = above median).

## Data Availability

The data used in this study are publicly available from the National Health and Nutrition Examination Survey (NHANES) program of the U. S. Centers for Disease Control and Prevention. All datasets can be accessed at https://www.cdc.gov/nchs/nhanes/index.htm. No additional data beyond these publicly available resources were generated or analyzed in this study.

## Appendix A. Supplementary data

Supplementary data to this article can be found online at https://doi.org/10.1016/j.maturitas.2026.108868.

## Abbreviations

5-MTHF: 5-methyltetrahydrofolate
BMI: Body Mass Index
CDC: Centers for Disease Control and Prevention
CI: Confidence Interval
COPD: Chronic Obstructive Pulmonary Disease
DNA: Deoxyribonucleic Acid
G1, G2, G3: MedHi index groups (none, below median, above median)
H–H; H–M; M–M; L–H; L–M; L–L: Dietary intake of live microbes combinations across two recall days
MeFox: 5-methyl-THF oxidation product
MedHi: Medium- and High-microbial Food Intake Index
LC–MS/MS: Liquid chromatography–tandem mass spectrometry
LOD: Limit of Detection
NHANES: National Health and Nutrition Examination Survey
NRF9.3: Nutrient-Rich Food Index 9.3
OR: Odds Ratio
RBC: Red Blood Cell
SAM: S-adenosylmethionine
SCFA: Short-Chain Fatty Acids
THF: Tetrahydrofolate
USDA: United States Department of Agriculture
