# Supplementary material for: Dietary intake of live microbes is inversely associated with fatigue and modified by serum folate among adults aged 40 years or more
Source: Maturitas. Author manuscript; Available in PMC 2026 Jun 30. (PMC13317025; doi:10.1016/j.maturitas.2026.108868)
Supplement: Supplementary [file NIHMS2186414-supplement-Supplementary.docx]

| **Supplementary Table 1. Definition and coding of sociodemographic, health, and dietary covariates used in NHANES 2011–2023 analyses**. | | | |
| --- | --- | --- | --- |
| **Covariate** | **NHANES variable(s)** | **Variable type** | **Coding / Derivation** |
| **Age** | RIDAGEYR | Continuous / Categorical | Recorded in years. Used as continuous in models and categorized as 20–39, 40–59, and ≥60 years for descriptive analyses. |
| **Sex** | RIAGENDR | Categorical | Male (1), Female (2). |
| **Race / Ethnicity** | RIDRETH1 | Categorical | Non-Hispanic White (3), Non-Hispanic Black (4), Other (includes Mexican American [1], Other Hispanic [2], and Other Race including multiracial [5]). |
| **Education** | DMDEDUC2 | Categorical | Less than high school (1–3), High school or GED (4), More than high school (5). |
| **Marital status** | DMDMARTZ / DMDMARTL | Categorical | Married or living with partner (1, 6), Formerly married (widowed, divorced, separated; 2–4), Never married (5). |
| **Body mass index (BMI)** | BMXBMI | Continuous / Categorical | Measured in kg m⁻²; categorized as <25.0 (normal weight), 25.0–29.9 (overweight), and ≥30.0 (obese). Participants <18.5 kg m⁻² (<5%) combined with the normal-weight group. |
| **Chronic disease indicator** | MCQ160b–f (cardiovascular conditions), MCQ160o/p (emphysema)*, MCQ220 (cancer), DIQ010 (diabetes) | Binary (Yes/No) | “Yes” = ≥1 self-reported physician diagnosis of congestive heart failure, coronary heart disease, angina, myocardial infarction, stroke, cancer, emphysema, or diabetes (any item = 1). “No” = none reported (all = 2). Missing = any item coded 9. |
| **Total energy intake** | DR1TKCAL, DR2TKCAL | Continuous / Categorical | Two-day mean intake (kcal day⁻¹). Used as continuous; categorized into tertiles for descriptive analyses. |
| **Total folate intake (food + supplements)** | DR1TFDFE, DR2TFDFE | Continuous | Average of day 1 and day 2 totals (µg DFE day⁻¹). Includes all folate forms from foods and supplements. |
| **Alcohol intake** | ALQ120Q, ALQ130, ALQ151 | Categorical | Harmonized across cycles. Non-drinker (never/past), Moderate (<2 drinks day⁻¹), or High (≥2 drinks day⁻¹). |
| **Diet quality (NRF 9.3 score)** | DR1TPROT, DR1TFIBE, DR1TVARA, DR1TVC, DR1TATOC, DR1TCALC, DR1TIRON, DR1TMAGN, DR1TPOTA; DR1TSFAT, DR1TSODI, DR1TSUGR | Continuous / Categorical | Nutrient-Rich Foods Index 9.3 per 100 kcal = Σ %DV of nine nutrients to encourage (each capped at 100) − %DV of saturated fat, sodium, and added sugars. Categorized into tertiles (T1–T3) for descriptive analyses. |
| **Fatigue** | DPQ040 | Binary | Derived from PHQ-9 item 4 (“Feeling tired or having little energy”). Responses coded as 0 = “not at all” or “several days” (0–1) and 1 = “more than half the days” or “nearly every day” (2–3). |
| **Core depressive symptoms** | DPQ010, DPQ020, DPQ060–DPQ090 | Binary | Constructed from PHQ-9 affective and cognitive items: item 1 (“little interest or pleasure”), item 2 (“feeling down or hopeless”), and items 6–9 (“feeling bad about yourself,” “trouble concentrating,” “moving/speaking slowly or too fast,” “thoughts of self-harm”). Items were summed and dichotomized as 0–1 versus ≥2. |
| **Sleep disturbance** | DPQ030 | Binary | Derived from PHQ-9 item 3 (“trouble sleeping or sleeping too much”). Responses summed and dichotomized as 0–1 versus ≥2. |

**Supplementary Material**

**Note.** Abbreviations: BMI, body mass index; NRF9.3, Nutrient-Rich Food Index 9.3; DFE, dietary folate equivalents; MEC, Mobile Examination Center.

| **Supplementary Table 2. Weighted characteristics of participants included in the complete-case analytic sample compared with excluded cases, NHANES 2011–2023** | | | | |
| --- | --- | --- | --- | --- |
|  | **Total** 23,342 (100.0%) | **Excluded Cases** 8,966 (23%) | **Complete Cases** 14,376 (77%) | **p-value** |
| **Characteristics** |  |  |  |  |
| **Age, years, mean** ±**SD** | 58.7± 11.5 | 60.3± 11.5 | 58.3± 11.5 | <0.001 |
| **Sex** |  |  |  | 0.165 |
| Male | 11,120 (47.3) | 4,35(49.0) | 6,770 (46.8) |  |
| Female | 12,222 (52.7) | 4,616 (51.1) | 7,606 (53.2) |  |
| **Race/Ethnicity** |  |  |  | 0.030 |
| NH White | 10,294 (60.2) | 3,596 (66.6) | 6,698 (70.0) |  |
| NH Black | 4,970 (10.6) | 2,053 (12.4) | 2,917 (10.0) |  |
| Others | 8,078 (20.2) | 3,317 (21.0) | 4,761 (20.0) |  |
| **Education** |  |  |  | 0.152 |
| Less than HS | 10,447 (36.5) | 4,422 (35.6) | 6,025 (35.6) |  |
| Education (HS Grad) | 6,718 (30.0) | 2,435 (30.7) | 4,283 (30.7) |  |
| More than HS | 6,129 (33.5) | 2,061 (33.8) | 4,068 (33.8) |  |
| **Marital Status** |  |  |  | <0.001 |
| Married/living with partner | 13,780 (66.9) | 5,027 (65.8) | 8,753 (67.2) |  |
| Formerly | 8,494 (29.4) | 3,652 (32.3) | 4,842 (28.6) |  |
| Never | 1,030 (3.7) | 249 (1.9) | 781 (4.2) |  |
| **BMI (kg/m^2^)**† |  |  |  | 0.282 |
| Normal | 5,183 (23.8) | 1,803 (25.6) | 3,380 (23.3) |  |
| Overweight | 6,994 (34.4) | 2,110 (33.9) | 4,884 (34.5) |  |
| Obese | 8,651 (41.8) | 2,539 (40.5) | 6,112 (42.7) |  |
| **Chronic Disease** |  |  |  |  |
| ≥1 of the above (Yes) | 9,678 (38.5) | 3,938 (44.0) | 5,740 (36.6) | <0.001 |
| **Diet Quality (**NRF9.3**)** |  |  |  | 0.088 |
| T1-Low | 4,628 (29.4) | 323 (28.8) | 4,305 (29.5) |  |
| T2-Medium | 5,320 (34.1) | 438 (38.4) | 4,882 (33.8) |  |
| T3-High | 5,613 (36.5) | 424 (32.8) | 5,189 (36.7) |  |
| **Energy Intake (Kcal)** | 2015±767 | 2013 ± 868 | 2016 ± 732 | 0.921 |
| **Total Folate Intake (µg/day)** | 480±270 | 467±288 | 483±266 | 0.052 |
| **Alcohol Intake** |  |  |  | 0.915 |
| Non-drinker (never/past) | 7,506 (30.8) | 2,031 (31.4) | 5,475 (30.7) |  |
| Moderate (<2 drinks day) | 4,616 (27.5) | 1,128 (27.3) | 3,488 (27.6) |  |
| High (≥2 drinks day) | 7,115 (41.7) | 1,702 (41.3) | 5,413 (41.7) |  |
| **Dietary live-microbe intake** |  |  |  | 0.219 |
| L–L | 3,819 (15.5) | 1,051 (17.2) | 2,768 (15.0) |  |
| L-M | 3,771 (20.2) | 891 (20.8) | 2,888 (20.0) |  |
| L-H | 4,458 (20.5) | 1,057 (20.8) | 3,401 (20.3) |  |
| M–M | 1,667 (10.9) | 334 (9.5) | 1,333 (11.3) |  |
| M-H | 2,785 (18.7) | 566 (17.3) | 2,219 (19.1) |  |
| H–H | 2,250 (14.4) | 475 (14.4) | 1,775 (14.3) |  |
| **MedHi Index** |  |  |  | 0.190 |
| G1 | 3,819 (15.5) | 1,051 (17.1) | 2,768 (15.0) |  |
| G2 (>0 & < median) | 6,653 (35.4) | 1,541 (36.0) | 5,112 (35.2) |  |
| G3 (≥ median) | 8,278 (49.1) | 1,782 (46.9) | 6,496 (49.8) |  |
| **Fatigue** |  |  |  | 0.913 |
| None/low | 15,712 (83.6) | 3,836 (83.5) | 11,876 (83.7) |  |
| Moderate-Severe | 3,408 (16.4) | 908 (16.6) | 2,500 (16.3) |  |

**Note.** Values are survey-weighted and expressed as percentages for categorical variables or means ± SD for continuous variables. p-values were derived from survey-adjusted Wald or Rao–Scott χ² tests as appropriate. Abbreviations: NH, non-Hispanic; HS, high school; BMI, body mass index; NRF9.3, Nutrient-Rich Food Index 9.3. **†** Normal weight defined as BMI 18.5–24.9 kg/m²; participants <18.5 kg/m² (<5% of sample) were included in this category.

| **Supplementary Table 3. Survey-weighted distributions of dietary intake of live microbes by fatigue status among U.S. adults aged ≥ 40 years** | | | | | | | | |
| --- | --- | --- | --- | --- | --- | --- | --- | --- |
|  | | |  | **Fatigue** | | |  |  |
|  |  | **Total** N (%) 14,376 (100.0) |  | **None/low** N (%) 11,681 (83.6) |  | **Moderate/Severe** N (%) 2,486 (16.4) |  | p-value |
| **Dietary intake of live microbes**† |  |  |  |  |  |  |  | < 0.001 |
| L-L |  | 2,768 (15.0) |  | 2,157 (13.7) |  | 611 (21.5) |  |  |
| L-M |  | 2,888 (20.0) |  | 2,338 (19.5) |  | 542 (22.3) |  |  |
| L-H |  | 3,401 (20.3) |  | 2,864 (20.6) |  | 537 (18.8) |  |  |
| M-M |  | 1,333 (11.3) |  | 1,076 (11.4) |  | 257 (10.7) |  |  |
| M-H |  | 2,219 (19.1) |  | 1,906 (19.7) |  | 313 (16.2) |  |  |
| H-H |  | 1,775 (14.3) |  | 1,535 (15.1) |  | 240 (10.5) |  |  |
| **MedHi Index**†† |  |  |  |  |  |  |  | < 0.001 |
| **G1** |  | 2,768 (15.0) |  | 2,157 (13.7) |  | 611 (21.5) |  |  |
| **G2 (>0 & < median)** |  | 5,112 (35.2) |  | 4,143 (34.8) |  | 969 (37.5) |  |  |
| **G3 (≥ median)** |  | 6,496 (49.8) |  | 5,576 (51.5) |  | 920 (41.0) |  |  |
| **Note.** Values are survey-weighted and expressed as percentages. p-values were obtained from survey-adjusted Wald or rank-sum tests comparing fatigue groups. **†** Live-microbiota intake categories (L–L, L–M, L–H, M–M, M–H, H–H) represent all combinations of low (L), medium (M), and high (H) microbial-rich food intake across two 24-hour dietary recalls. **††** MedHi Index groups (G1–G3) reflect total gram intake of medium- and high-microbial foods: G1 = none; G2 = >0 and < median; G3 = ≥ median. | | | | | | | | |

| **Supplementary Table 4. Survey-weighted distribution of serum folate metabolites by fatigue status among U.S. adults aged ≥ 40 years** | | | | | | | | |  |
| --- | --- | --- | --- | --- | --- | --- | --- | --- | --- |
|  |  |  |  | **Fatigue** | | |  |  |  |
|  |  | **Total** N=14,376 |  | **None/low** N=11,681 |  | **Moderate/Severe**  N=2,486 |  | p-value |  |
| **Serum Folate Metabolites (**nmol/L) |  |  |  | **Median [IQR]** | | |  |  |  |
| RBC |  | 521[$401\text{–}698$] |  | 521[405-698] |  | 521[390–720] |  | 0.189 |  |
| Total folate |  | 39.7[25.9–59.2] |  | 40.1[26.3–59.7] |  | 36.8[23.6–56.6] |  | **0.060** |  |
| 5-methyl-THF |  | 37.2[24.0–55.7] |  | 37.8[24.5–56.3] |  | 34.2[21.8–52.8] |  | **0.008** |  |
| Folic acid |  | 0.68[0.48–1.07] |  | 0.68[0.48–1.06] |  | 0.70[0.50–1.08] |  | **0.075** |  |
| THF |  | 0.14[0.14–0.14] |  | 0.14[0.14–0.14] |  | 0.14[0.14–0.14] |  | 0.598 |  |
| 5-formyl-THF / folinic acid |  | 0.84[0.56–1.20] |  | 0.84[0.56–1.20] |  | 0.85[0.55–1.21] |  | 0.737 |  |
| 5,10-methenyl-THF |  | 0.14[0.14–0.22] |  | 0.14[0.14–0.22] |  | 0.14[0.14–0.22] |  | 0.579 |  |
| MeFox (oxidized) |  | 1.57[0.97–2.56] |  | 1.54[0.96–2.53] |  | 1.67[1.00–2.76] |  | **<0.001** |  |
| **Note.** Values are survey-weighted and shown in original (pre–log-transformed) units as medians with interquartile ranges. p-values were derived from survey-weighted linear regression models in which each log-transformed folate metabolite was regressed on fatigue status. Serum total folate represents the sum of all measured metabolites (5-methyl-THF, folic acid, THF, 5-formyl-THF, 5,10-methenyl-THF, and MeFox), quantified using LC–MS/MS. RBC folate was converted from ng/mL to nmol/L using 1 ng/mL = 2.266 nmol/L. Abbreviations: RBC, red blood cell; THF, tetrahydrofolate; MeFox, oxidized 5-methyl-THF; IQR, interquartile range | | | | | | | | |  |

| **Supplementary Table 5. Survey-weighted associations between dietary intake of live microbes and fatigue after adjustment for core depressive symptoms among U.S. adults aged ≥40 years** | | |
| --- | --- | --- |
| **Dietary intake of live microbes**† | **OR (95% CI)** | **p-value** |
| L-L | 1.00 | — |
| M–L | 0.79 (0.62–1.01) | 0.063 |
| H–L | 0.70 (0.55–0.89) | 0.004 |
| M–M | 0.63 (0.48–0.82) | 0.001 |
| H–M | 0.70 (0.53–0.94) | 0.017 |
| H–H | 0.64 (0.47–0.87) | 0.005 |
| p-trend |  | 0.002 |
| Overall P-value |  | 0.008 |
| **MedHi Index**†† |  |  |
| Low (reference) | 1.00 |  |
| Medium | 0.72 (0.59–0.88) | 0.002 |
| High | 0.71 (0.55–0.92) | 0.010 |
| p-trend |  | 0.030 |
| Overall P-value |  | 0.007 |
| Note. Values are survey-weighted odds ratios (ORs) and 95% confidence intervals (CIs) from logistic regression models. Core depressive symptoms were defined using affective and cognitive PHQ-9 items (items 1, 2, and 6–9) and dichotomized as 0–1 versus ≥2. Models were adjusted for age, sex, race/ethnicity, education, marital status, BMI category, chronic disease status, NRF9.3 diet quality score, alcohol intake, and total energy intake. p-values correspond to survey-adjusted Wald tests for linear trend or overall group differences. Given that fatigue prevalence was approximately 16.3%, ORs should be interpreted as measures of relative odds and may modestly overestimate corresponding risk ratios; they should not be interpreted as risk ratios or absolute measures of effect. † Categories (L–L, L–M, M–M, H–L, H–M, H–H) represent all combinations of low (L), medium (M), and high (H) microbial-rich food intake across two 24-hour dietary recalls. †† The MedHi Index provides an alternative semi-quantitative measure based on total gram intake of medium- and high-microbial foods (G1 = none, G2 = below median, G3 = above median among consumers). | | |

| **Supplementary Table 6. Survey-weighted associations between dietary intake of live microbes and fatigue after adjustment for sleep disturbance among U.S. adults aged ≥40 years** | | |
| --- | --- | --- |
| **Dietary intake of live microbes**† | **OR (95% CI)** | **p-value** |
| L-L | 1.00 | — |
| M–L | 0.84 (0.65–1.08) | 0.165 |
| H–L | 0.77 (0.59–0.99) | 0.040 |
| M–M | 0.68 (0.51–0.91) | 0.011 |
| H–M | 0.79 (0.59–1.05) | 0.099 |
| H–H | 0.62 (0.45–0.86) | 0.004 |
| p-trend |  | 0.004 |
| Overall P-value |  | 0.054 |
| **MedHi Index**†† |  |  |
| L-L | 1.00 |  |
| Medium | 0.79 (0.64–0.97) | 0.025 |
| High | 0.76 (0.59–0.96) | 0.025 |
| p-trend |  | 0.051 |
| Overall P-value |  | 0.048 |
| **Note.** Values are survey-weighted odds ratios (ORs) and 95% confidence intervals (CIs) from logistic regression models. Sleep disturbance was defined using PHQ-9 item 3 (*trouble sleeping or sleeping too much*) and dichotomized as 0–1 versus ≥2. Models were adjusted for age, sex, race/ethnicity, education, marital status, BMI category, chronic disease status, NRF9.3 diet quality score, alcohol intake, and total energy intake. *p*-values correspond to survey-adjusted Wald tests for linear trend or overall group differences. Given that fatigue prevalence was approximately 16.3%, ORs should be interpreted as measures of relative odds and may modestly overestimate corresponding risk ratios; they should not be interpreted as risk ratios or absolute measures of effect. † Categories (L–L, L–M, M–M, H–L, H–M, H–H) represent all combinations of low (L), medium (M), and high (H) microbial-rich food intake across two 24-hour dietary recalls. †† The MedHi Index provides an alternative semi-quantitative measure based on total gram intake of medium- and high-microbial foods (G1 = none, G2 = below median, G3 = above median among consumers). | | |
